# Kidney collecting duct cells make vasopressin in response to NaCl-induced hypertonicity

**DOI:** 10.1172/jci.insight.161765

**Published:** 2022-12-22

**Authors:** Juan Pablo Arroyo, Andrew S. Terker, Yvonne Zuchowski, Jason A. Watts, Fabian Bock, Cameron Meyer, Wentian Luo, Meghan E. Kapp, Edward R. Gould, Adam X. Miranda, Joshua Carty, Ming Jiang, Roberto M. Vanacore, Elizabeth Hammock, Matthew H. Wilson, Roy Zent, Mingzhi Zhang, Gautam Bhave, Raymond C. Harris

**Affiliations:** 1 Division of Nephrology and Hypertension, Department of Medicine, and; 2 Vanderbilt Center for Kidney Disease, Vanderbilt University Medical Center, Nashville, Tennessee, USA.; 3 Epigenetics and Stem Cell Laboratory, National Institute of Environmental Health Sciences, NIH, Research Triangle Park, North Carolina, USA.; 4 Division of Renal Pathology, Department of Pathology, Microbiology, and Immunology, Vanderbilt University Medical Center, Nashville, Tennessee, USA.; 5 Department of Pathology, Case Western Reserve University, University Hospitals, Cleveland, Ohio, USA.; 6 Vanderbilt University School of Medicine, Nashville, Tennessee, USA.; 7 Department of Psychology, Florida State University, Tallahassee, Florida, USA.; 8 Department of Veterans Affairs, Tennessee Valley Healthcare System, Nashville, Tennessee, USA.

**Keywords:** Nephrology, Epithelial transport of ions and water

## Abstract

Vasopressin has traditionally been thought to be produced by the neurohypophyseal system and then released into the circulation where it regulates water homeostasis. The questions of whether vasopressin could be produced outside of the brain and if the kidney could be a source of vasopressin are raised by the syndrome of inappropriate antidiuretic hormone secretion (vasopressin). We found that mouse and human kidneys expressed vasopressin mRNA. Using an antibody that detects preprovasopressin, we found that immunoreactive preprovasopressin protein was found in mouse and human kidneys. Moreover, we found that murine collecting duct cells made biologically active vasopressin, which increased in response to NaCl-mediated hypertonicity, and that water restriction increased the abundance of kidney-derived vasopressin mRNA and protein expression in mouse kidneys. Thus, we provide evidence of biologically active production of kidney-derived vasopressin in kidney tubular epithelial cells.

## Graphical abstract

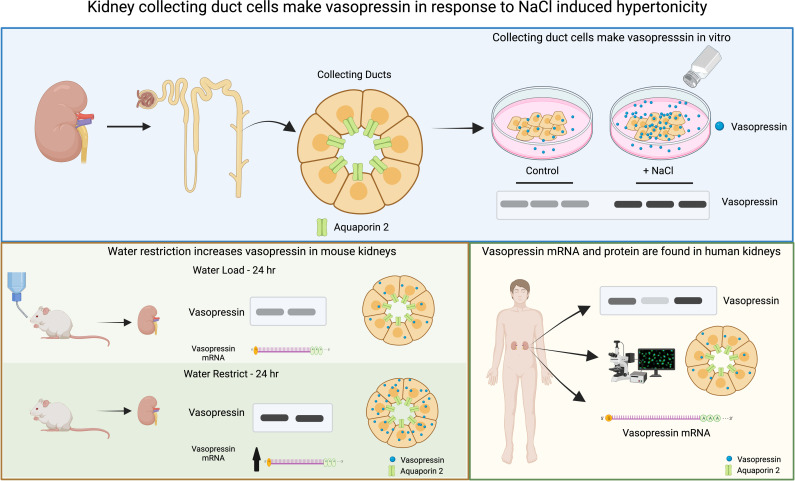

## Introduction

Vasopressin is a 9–amino acid peptide hormone that plays a key role in water and blood pressure homeostasis (1). Vasopressin is the end product of a highly processed 164–amino acid prepropeptide. Processing of the vasopressin prepropeptide results in 3 distinct peptides with a 1:1:1 ratio: vasopressin, neurophysin 2, and copeptin. Vasopressin is the biologically active hormone, neurophysin 2 is a carrier protein for vasopressin, and copeptin is the c-terminal glycosylated end product. Physiologic vasopressin production is currently thought to be limited to the brain under physiologic conditions. The main physiologic stimuli for vasopressin production in the hypothalamus are increased extracellular fluid tonicity and hypotension (1). Vasopressin binds to 3 distinct G-coupled protein receptors, V1a, V1b, and V2. The V2 receptor (V2R) is mainly expressed in the kidney along the distal nephron and is a critical regulator of mRNA transcription, protein abundance, and trafficking of the water channel, aquaporin 2 (AQP2) (2–4). Vasopressin signaling through V2R leads to AQP2 phosphorylation and translocation to the apical membrane of connecting and collecting duct cells (5), which leads to increased apical water permeability, with consequent water retention and increased urine concentration.

Unregulated vasopressin production can lead to excessive water retention and decreased serum sodium (Na^+^) levels. This common clinical scenario, known as syndrome of inappropriate antidiuretic hormone secretion, occurs in several diseases processes, including malignancy, pulmonary disorders, central nervous system disorders, and nonhypothalamic tumors, and with certain medications (6). Moreover, there is a large body of work that shows that vasopressin is involved in a broad range of physiologic and pathophysiologic states that go beyond water and blood pressure homeostasis (7–20). In fact, there are multiple reports of local vasopressin mRNA synthesis outside of the brain (21–29). This raises the questions of whether biologically active vasopressin is also produced outside of the brain under physiologic conditions and if this locally produced vasopressin functions independent of pathways linked to water transport and body fluid balance.

To answer these questions, we investigated whether kidney epithelial cells produce vasopressin and if this production is regulated by changes in extracellular fluid tonicity. We found evidence of vasopressin gene expression and protein production in mouse and human kidney epithelial cells. We demonstrated that this vasopressin activated V2R in vitro, and its production was increased when cells were placed in hypertonic NaCl solution. Finally, we provide evidence that whole-kidney vasopressin mRNA and protein expression increased in water-deprived mice. Thus, we conclude that kidney epithelial cells produce vasopressin that can be increased by NaCl-mediated hypertonicity under physiological conditions.

## Results

### Vasopressin mRNA is present in human and mouse kidneys.

**Figure 1:**
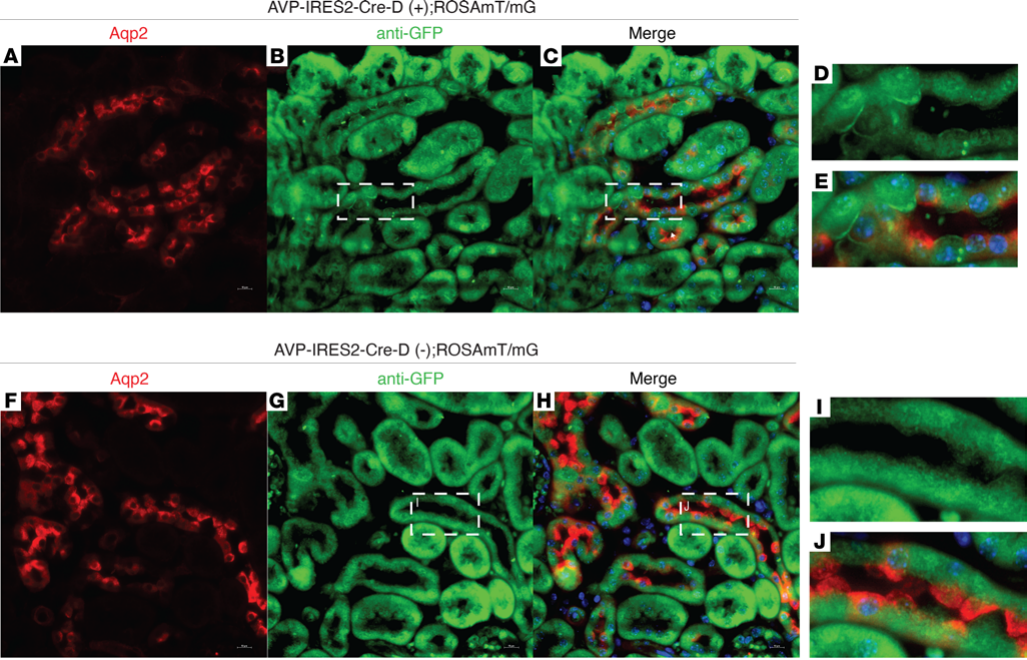
*Avp* is expressed in mouse collecting duct cells in vivo. When crossed with a Rosa26mT/mG reporter mouse (mTmG), (**A–E**) mice expressing Cre recombinase linked to AVP gene expression (AVP-IRES-Cre-D(+); mTmG) express membrane GFP, (**F–J**) while mice lacking AVP-Cre (AVP-IRES-Cre-D(–); mTmG) do not. Boxed regions in **B** and **C** as well as **G** and **H** are shown at higher magnification in **D** and **E** as well as **I** and **J**, respectively. Original magnification, ×60 (**A**–**C** and **F**–**H**); ×100 (**D**, **E**, **I**, and **J**).

We initially interrogated the Genotype-Tissue Expression Project database to identify potential sites of extrahypothalamic vasopressin production in humans (30). Vasopressin (*Avp*) mRNA expression was highest in the hypothalamus, followed by testis, kidney cortex, and kidney medulla (Supplemental Figure 1A; supplemental material available online with this article; https://doi.org/10.1172/jci.insight.161765DS1). We confirmed the database-defined expression of *Avp* mRNA in mouse brain and kidney tissue by performing RT-PCR on various tissues (Supplemental Figure 1B). We also found that vasopressin mRNA was present in principal cells of mice utilizing the mouse kidney single-cell RNA-Seq data set, available through the Kidney Interactive Transcriptomics website (http://humphreyslab.com/SingleCell) (Supplemental Figure 1C). We used the NephroSeq Application, available through the University of Michigan (31), to analyze publicly available, peer-reviewed data sets, which include both RNA-Seq and microarray data, and found 22 analyses in which Avp gene expression was reported (Supplemental Tables 1 and 2). To confirm these observations, we created a reporter mouse, AVP-IRES2-Cre-D;ROSA^mT/mG^, in which Cre-recombinase expression linked to the vasopressin gene drives expression of membrane GFP. Cells with membrane GFP (mGFP) either actively express or are derived from cells that expressed the vasopressin gene. We found that mGFP was present in principal and intercalated cells in AVP Cre (+) mice (Figure 1, A–E) but not in AVP Cre (–) mice (Figure 1, F–J). We then analyzed single-cell RNA-Seq data from rat primary inner medullary collecting duct cells (IMCDs) (32) and found that rat primary IMCDs in culture expressed *Avp* mRNA at levels comparable to *Aqp2*, *Aqp3*, *Aqp4*, *Scnn1a*, *Slc14a2*, and *Avpr2* (Supplemental Figure 2, A and B). Together, these data suggest that the vasopressin gene is expressed in the kidney and that collecting duct cells likely express *Avp* mRNA at baseline.

### Hypertonicity regulates Avp mRNA expression in collecting duct cells in vitro.

**Figure 2:**
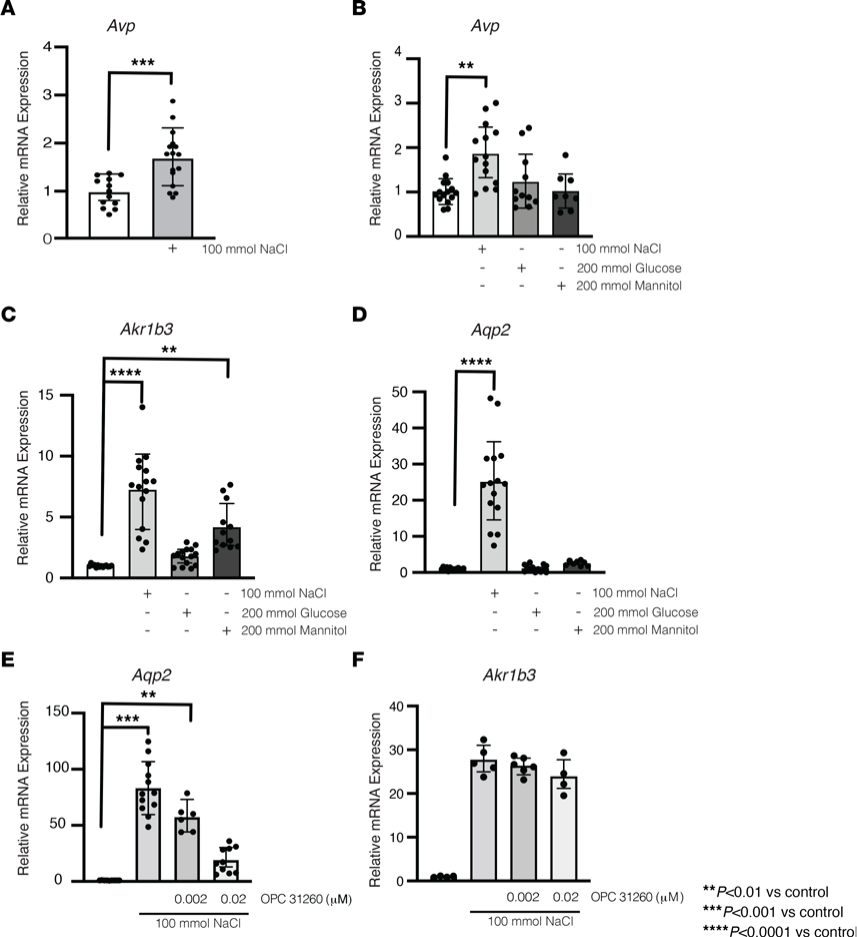
NaCl-mediated hypertonicity increased vasopressin mRNA in kidney epithelial cells. (**A**) Incubation with NaCl (500 mOsm) for 24 hours increased vasopressin (*Avp*) mRNA in mouse inner medullary collecting duct cells (*P* < 0.001 vs. control). (**B**) NaCl (*P* < 0.01) but not mannitol or glucose increase *Avp* mRNA. (**C**) Aldose reductase (*Akr1b3*), a marker of hypertonic stress, increased with NaCl (*P* < 0.0001 vs. control) and mannitol (*P* < 0.001 vs. control) but not glucose. (**D**) Aquaporin 2 (*Aqp2*) mRNA increased after treatment with NaCl but not glucose or mannitol. (**E**) Vasopressin receptor 2 antagonist OPC-31260 (20 nm) blunted the NaCl-mediated increase in *Aqp2*, (**F**) despite similar hypertonic stress. Data are presented as mean ± SD and analyzed with (**A**) an unpaired Mann-Whitney or (**B–F**) 1-way ANOVA with Tukey’s post hoc analysis, with a minimum of 4 independent replicates per group. **P < 0.01, ***P < 0.001, ****P < 0.0001 vs. control.

Because the single-cell RNA-Seq data suggested that there was vasopressin expression in collecting duct cells in vivo, we performed quantitative RT-PCR to explore whether *Avp* mRNA was present in mouse collecting duct cells in vitro. Vasopressin mRNA was detectable at baseline and increased when the cells were exposed to DMEM supplemented with 100 mmol NaCl (Figure 2A and Supplemental Figure 2D). There was no statistically significant change to vasopressin mRNA expression after 12 hours in NaCl-supplemented DMEM or 24 hours in hypotonic DMEM (Supplemental Figure 2, E and F). To determine whether increased vasopressin mRNA expression was due to increased concentration of NaCl per se or was a response to the increased hypertonicity of the medium, we added either 100 mmol NaCl or 200 mmol mannitol or glucose to the DMEM medium. The increased *Avp* transcription was only seen in the presence of NaCl supplementation, suggesting the effect was specific to the NaCl stimulation (Figure 2B). In contrast, expression of aldose reductase (*Akr1b3*), which is sensitive to increased extracellular tonicity, increased significantly in response to both NaCl and mannitol (Figure 2C) (33). Because *Aqp2* expression increases with activation of the V2r and hypertonicity (34), we measured the expression of this gene in response to NaCl, glucose, or mannitol and found that only DMEM supplemented with 100 mmol NaCl increased its expression (Figure 2D). This increase was likely due to autocrine or paracrine vasopressin production by the IMCDs, because *Aqp2* expression was significantly inhibited by addition of the nonpeptide V2r antagonist OPC-31260 (35, 36) (Figure 2E). In contrast, *Akr1b3* expression induced by NaCl was not affected by the V2r antagonist (Figure 2F), suggesting that there was similar hypertonic stress between groups and that the effect of the drug was specific. Taken together, these results indicate that cultured IMCDs produce vasopressin mRNA, which is regulated by NaCl-induced hypertonicity. In addition, they suggest that IMCDs produce locally active vasopressin that can regulate *Aqp2* expression in vitro.

### Development of an antibody that detects locally produced preprovasopressin.

**Figure 3:**
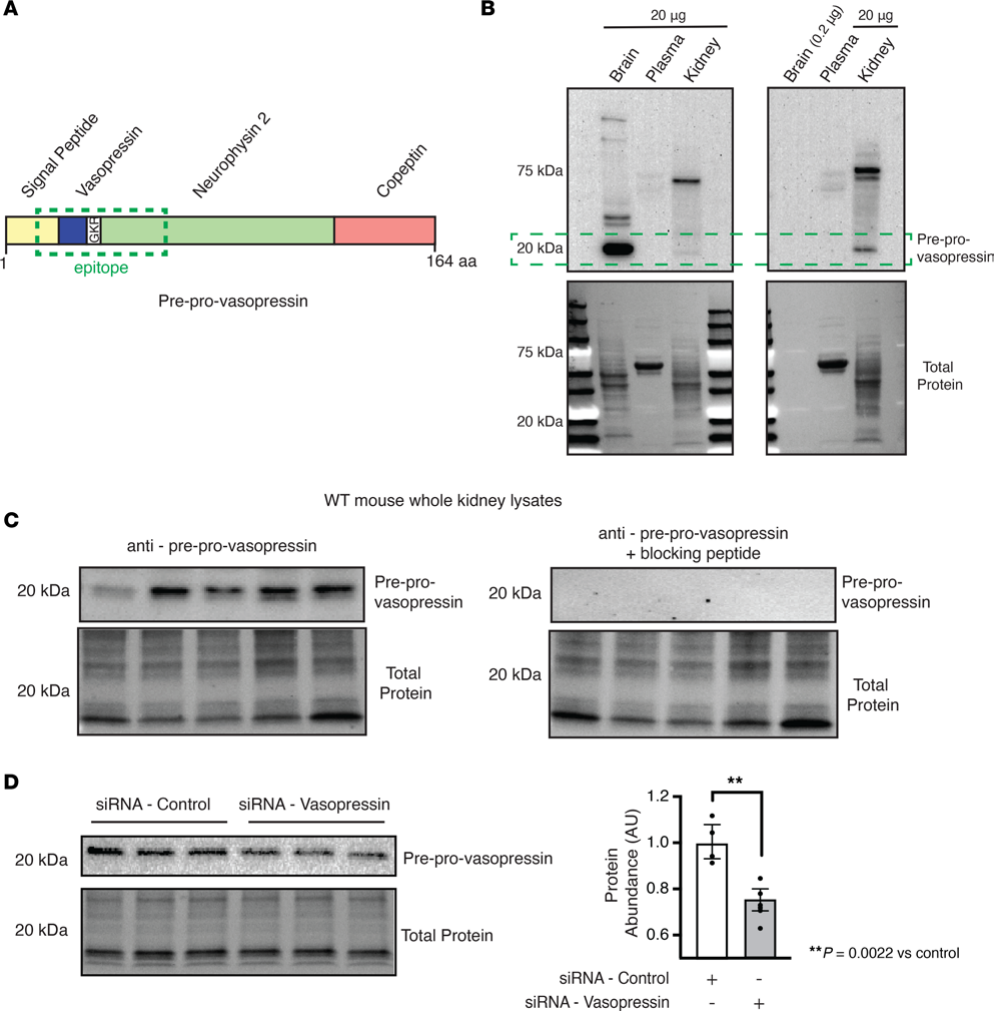
Preprovasopressin was found in mouse brain and kidney. (**A**) Our custom antibody targets the cleavage site of the vasopressin precursor peptide. (**B**) The antibody detected a specific band at 20 kDa in mouse brain and kidney samples, but not in plasma, at a 1:1 ratio (left) and a 1:10 ratio (right). (**C**) We detected a specific band in WT mouse kidneys (*n* = 5), and the signal was inhibited if the primary antibody was incubated with the blocking peptide. (**D**) Vasopressin was found in inner medullary collecting duct cells, and expression was decreased with the use of siRNA that targets *Avp*. Data in **D** are presented as mean ± SD and analyzed with an unpaired Mann-Whitney test; *n* = 4 independent replicates. **P = 0.002.

We next set out to confirm that kidney collecting ducts cells produced biologically active vasopressin. To do this we needed to develop tools, because vasopressin protein is difficult to quantify. Currently, vasopressin is quantified by an ELISA assay for copeptin, which is used as a surrogate for vasopressin levels (37). Moreover, the epitopes for the commercially available antibodies target end products of preprovasopressin processing, making it impossible to distinguish the site of production from peripheral tissue uptake. To confirm that kidney collecting duct cells produced biologically active vasopressin, we developed a custom antibody in which the epitope spans the known cleavage sites for the vasopressin precursor peptide (Figure 3A), which allowed us to measure locally produced preprovasopressin (38). We characterized the antibody by performing a Western blot on the brain from a WT mouse in the presence and absence of the blocking peptide. The antibody detected an intense band at the expected weight for preprovasopressin (~20 kDa) in the brain, and the signal was abolished with the blocking peptide (Supplemental Figure 3A). We then confirmed the antibody did not detect the 9–amino acid variants of vasopressin or oxytocin (Supplemental Figure 3B). Immunostaining of mouse brain sections showed an intense signal in the supraoptic nucleus, containing vasopressin-producing cell bodies, and the internal layer of the median eminence, containing axons of vasopressin-producing neurons that project to the posterior pituitary (Supplemental Figure 3C) (39). To test the specificity of our antibody in cells, we transfected HEK293 cells with a vasopressin expressing vector (pCMV6-XL-5-*AVP*) and found a single band at the expected weight for preprovasopressin (Supplemental Figure 3D). We further characterized the antibody by performing a Western blot on brain, kidney, and plasma. The antibody detected an intense band at the expected weight for preprovasopressin (~20 kDa) in the brain and the kidney (Figure 3B) but not in the plasma. To confirm the presence of preprovasopressin in the kidneys in vivo, we obtained WT mouse whole-kidney lysates and incubated them with anti-preprovasopressin primary antibody, with and without incubation with the blocking peptide. We detected a band of the expected weight, and the signal was eliminated by the blocking peptide (Figure 3C). To verify that IMCDs produced preprovasopressin, we performed immunoblots on cell lysates. We saw the expected single 20 kDa band that decreased when the IMCDs were transfected with *Avp* siRNA (Figure 3D). Immunoprecipitation from whole-cell IMCD lysates using our potentially novel antibody identified a single band at the expected weight for preprovasopressin (Supplemental Figure 3, E and F). Additionally, immunostaining of our immunoprecipitation sample with commercially available antibodies that target the 9–amino acid vasopressin and neurophysin-2 showed a band at the expected weight for preprovasopressin (Supplemental Figure 3, G and H). Thus, we had produced a highly specific antibody that could identify uncleaved preprovasopressin.

### IMCDs produce biologically active vasopressin.

**Figure 4:**
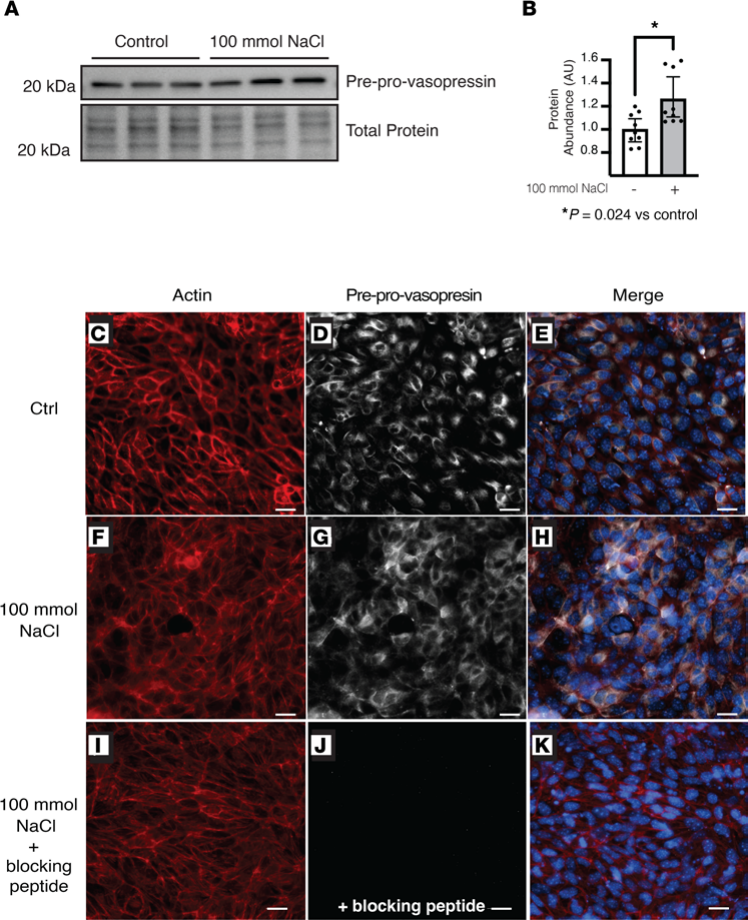
NaCl increased preprovasopressin protein in inner medullary collecting duct cells. Treatment of inner medullary collecting duct cells with NaCl for 24 hours increased the abundance of vasopressin by (**A** and **B**) immunoblot and (**C–K**) immunofluorescence. (**I–K**) Preincubation of the primary antibody with the blocking peptide abolished the signal. Data in **B** are presented as mean ± SD and analyzed with an unpaired Mann-Whitney test; *n* = 9 independent replicates. *P = 0.024. Scale bar: 10 μM.

To assess if preprovasopressin protein increased in the same conditions as vasopressin mRNA, we stimulated IMCDs with NaCl for 24 hours and determined that vasopressin precursor protein expression increased indeed (Figure 4, A and B). Immunofluorescence staining of IMCDs stimulated with NaCl also showed increased staining for vasopressin (Figure 4, G and H) versus controls (Figure 4, D and E), and the signal was once again abolished with the blocking peptide (Figure 4, J and K; see Figure 4, C, F, and I, for actin controls).

**Figure 5:**
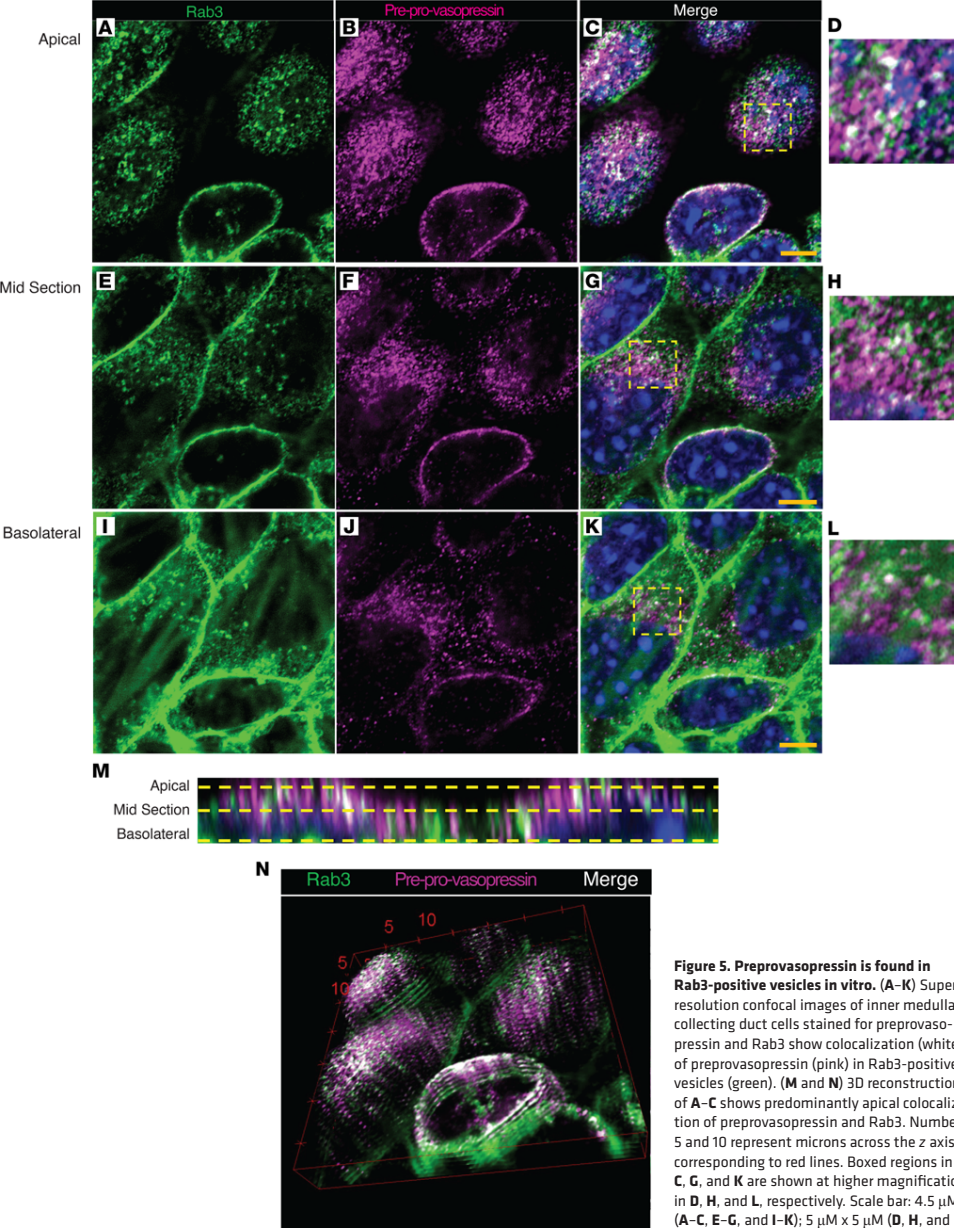
Preprovasopressin is found in Rab3-positive vesicles in vitro. (**A**–**K**) Superresolution confocal images of inner medullary collecting duct cells stained for preprovasopressin and Rab3 show colocalization (white) of preprovasopressin (pink) in Rab3-positive vesicles (green). (**M** and **N**) 3D reconstruction of **A**–**C** shows predominantly apical colocalization of preprovasopressin and Rab3. Numbers 5 and 10 represent microns across the *z* axis, corresponding to red lines. Boxed regions in **C**, **G**, and **K** are shown at higher magnification in **D**, **H**, and **L**, respectively. Scale bar: 4.5 μM (**A**–**C**, **E**–**G**, and **I**–**K**); 5 μM x 5 μM (**D**, **H**, and **L**).

In neurons, vasopressin is stored in secretory vesicles. Therefore, to evaluate whether preprovasopressin was found in secretory vesicles in the kidney, we stained IMCDs for preprovasopressin and Rab3 and performed imaging with superresolution confocal microscopy (Figure 5). We found that some, but not all, preprovasopressin was colocalized with Rab3 (Figure 5, A–L), and a composite 3D image of the cells suggested apical enrichment of Rab3-positive preprovasopressin-containing vesicles (Figure 5, M and N). These data suggest that preprovasopressin in IMCDs is found in Rab3-positive secretory vesicles.

**Figure 6:**
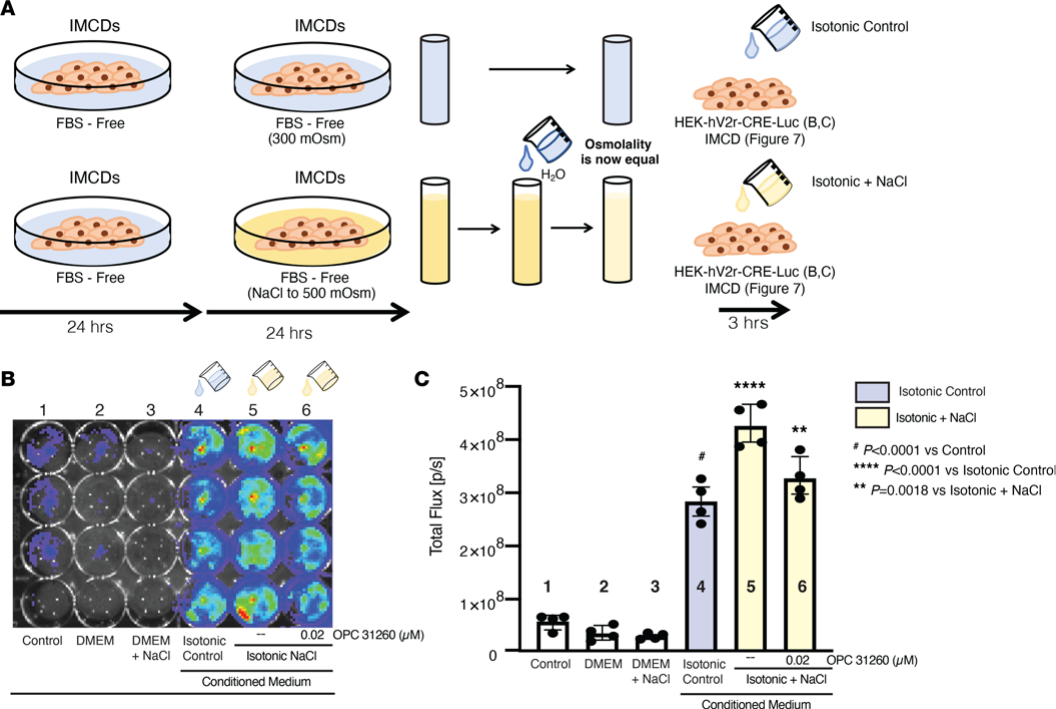
Inner medullary collecting duct cell medium contains a V2R-stimulating ligand. (**A**) Bioassay experimental design to obtained conditioned medium from treated and untreated inner medullary collecting duct cells (IMCDs). (**B** and **C**) HEK-V2R-Luc cells stimulated with control and NaCl-treated IMCD medium had increased luciferase activity (lanes 4 and 5). The NaCl-mediated increase was blocked with the V2R antagonist OPC-31260 (lane 6). Data in **C** are presented as mean ± SD of total flux (photons per second). Data were analyzed with 1-way ANOVA with Tukey’s post hoc analysis, with a minimum of 4 independent replicates per group. ^#^*P* < 0.0001 vs. control; ****P < 0.0001 vs. isotonic control; **P = 0.0018 vs. isotonic + NaCl.

To confirm that the vasopressin produced by the IMCDs was biologically active, we developed a cell line: stably transfected HEK293 cells with the human V2R (hV2R) and a cAMP response element driving luciferase expression (HEK-hV2R-CRE-Luc) that can be activated by V2R activation. We then developed a bioassay in which we collected medium from control or NaCl stimulated IMCDs, as illustrated in Figure 6A. Serum-starved HEK-hV2R-CRE-Luc cells were then subjected to the following: (a) no medium change, (b) fresh FBS-free DMEM, (c) fresh FBS-free DMEM plus 100 mmol NaCl, (d) IMCD control medium, (e) IMCD NaCl medium, and (f) IMCD NaCl medium plus OPC-31260 (Figure 6B). We found that medium from IMCDs stimulated luciferase production. The luciferase activity was higher in media supplemented with NaCl than IMCD control medium, and the increased activity was abolished with the addition of OPC-31260 (Figure 6C). Together, these data suggest that there is biologically active vasopressin in IMCD-conditioned medium at baseline, which increases after NaCl treatment, and the NaCl-dependent increase in luciferase activity was due to V2R activation, as it was prevented with V2R antagonist OPC-31260.

**Figure 7:**
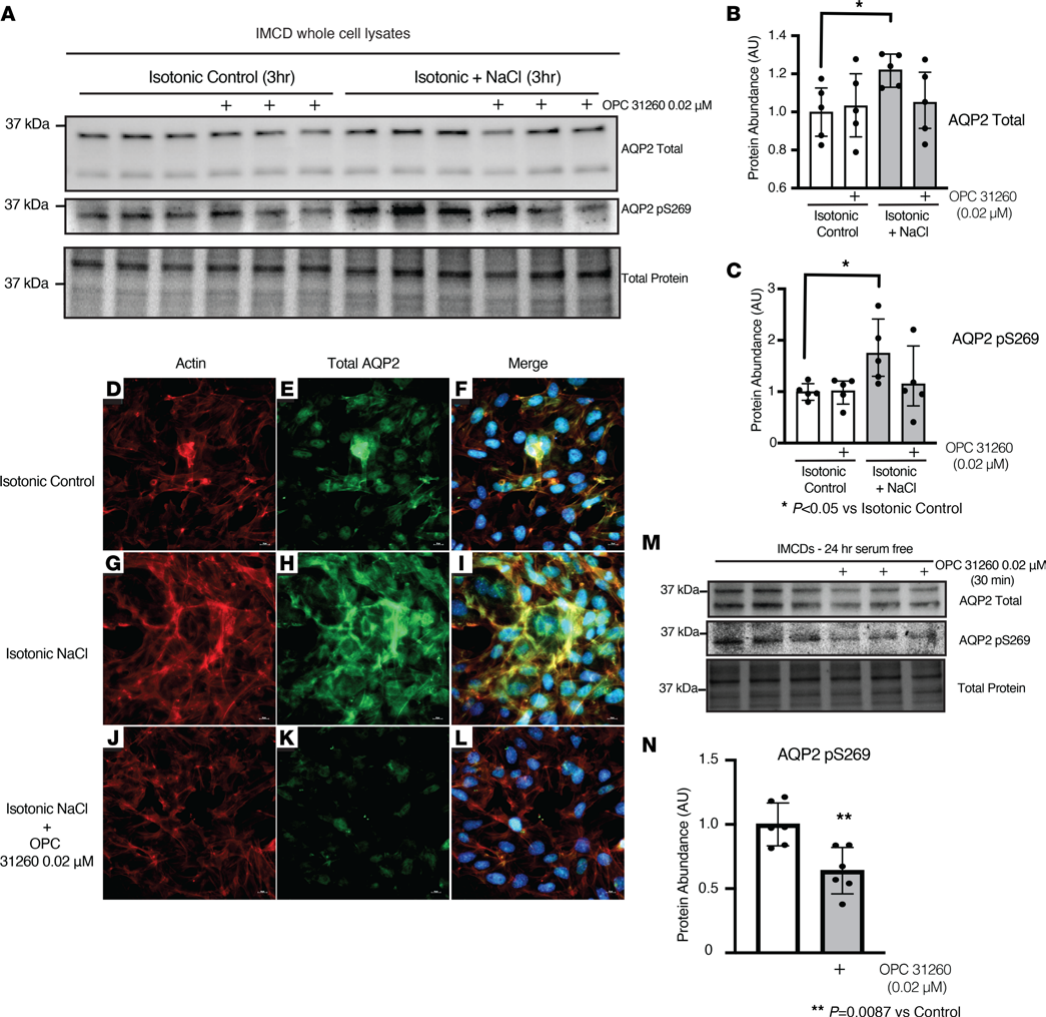
Inner medullary collecting duct cell medium ligand modifies AQP2 expression and phosphorylation. (**A–C**) In inner medullary collecting duct cells (IMCDs) addition of isotonic and NaCl-conditioned medium increased both phosphorylation of serine 269 and total abundance of AQP2, and this effect was prevented with OPC-31260. (**E** and **F** vs. **H** and **I**) Isotonic and NaCl-treated medium increased the staining of aquaporin 2 (AQP2) (green), relative to isotonic control medium. (**K** and **L**) The increase was prevented with OPC-31260. (**M** and **N**) Treatment of serum-starved IMCDs with OPC-31260 decreased phosphorylation of AQP2 serine 269. (**D**, **G**, and **J**) Actin controls. Data were analyzed with 1-way ANOVA with Tukey’s post hoc analysis, with a minimum of 4 independent replicates per group. *P < 0.05 vs. isotonic control. Original magnification, ×600.

We further confirmed the presence of biologically active vasopressin in IMCD-conditioned medium by adding control-conditioned medium or NaCl-supplemented conditioned medium (Figure 6A) to serum-starved IMCDs (Figure 7). We then assessed the expression and phosphorylation of AQP2 (Figure 7). As expected, when serum-starved IMCDs were treated with conditioned medium from IMCDs exposed to DMEM and 100 mmol NaCl, expression and phosphorylation of AQP2 increased (Figure 7, A–C and D–F vs. G–I), and this increase was blocked by the addition of OPC-31260 (Figure 7, D–F vs. J–L). To directly evaluate V2r activation by locally produced vasopressin, fresh FBS-free DMEM was added to 24 serum-starved IMCDs, followed by treatment with vehicle or V2r antagonist OPC-31260 for 30 minutes. Treatment with OPC-31260 decreased AQP2 phosphorylation at baseline (Figure 7, M and N). This suggests that baseline V2r activation in serum-starved IMCDs is dependent on local vasopressin production, which can be antagonized by treatment with a V2r antagonist. Together, these data suggest that IMCDs make biologically active vasopressin that increases when stimulated with NaCl, and local vasopressin can regulate AQP2 total protein abundance and phosphorylation.

### Vasopressin mRNA and protein increased with water restriction in vivo.

**Figure 8:**
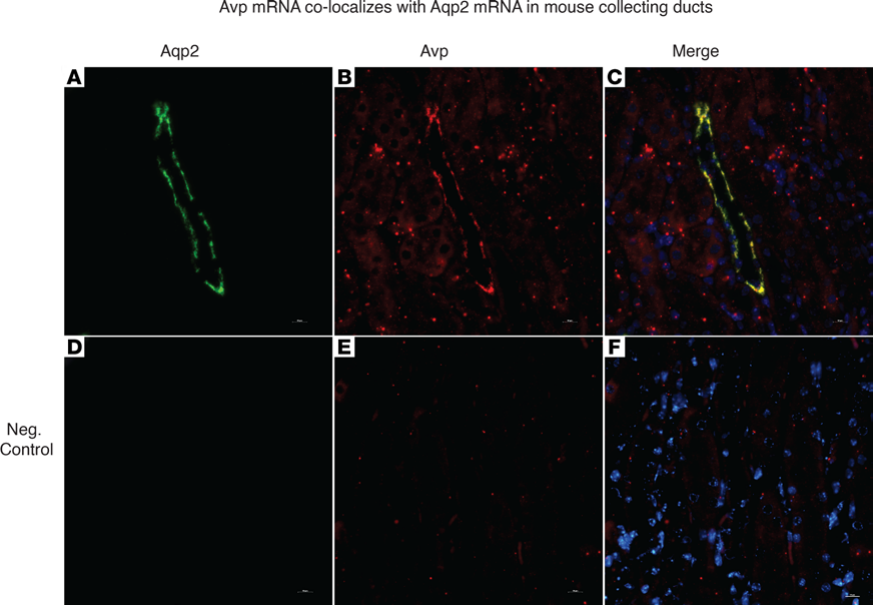
Vasopressin mRNA is found in mouse collecting duct cells in vivo. RNA in situ hybridization (RNAScope) in mouse kidneys showed colocalization of (**A**) Aqp2 and (**B**) vasopressin mRNA in (**C**) collecting ducts. (**D–F**) Negative control. Scale bar: 5 μM.

To confirm expression of vasopressin by the kidneys in vivo, we looked for evidence that vasopressin mRNA is made by collecting ducts in mice. We used RNA in situ hybridization (RNAScope) and found that *Avp* mRNA colocalized with *Aqp2* mRNA in mouse kidneys (Figure 8, A–C) versus negative control (Figure 8, D–F). This supported the publicly available RNA-Seq data that suggested expression of *Avp* mRNA by collecting duct cells in vivo (Supplemental Figure 2).

**Figure 9:**
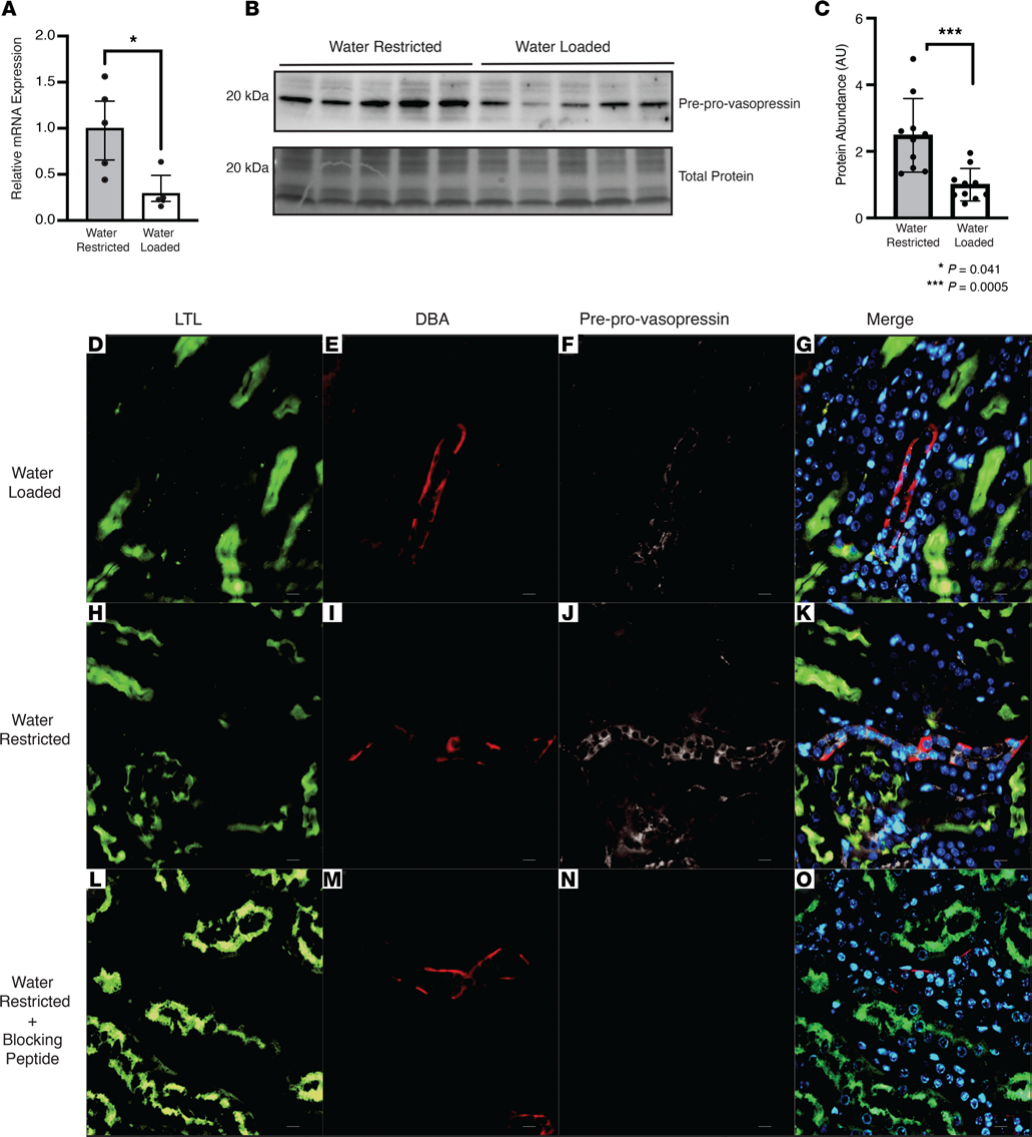
Kidney-derived vasopressin increases after water restriction. (**A**) *Avp* mRNA increased in water-restricted mice relative to water-loaded controls. (**B** and **C**) Kidney-derived preprovasopressin protein increased after water restriction relative to water loading. (**D–O**) In mouse kidney, kidney-derived preprovasopressin signal (white) in collecting ducts (DBA, red) increased in water-restricted animals (**J** vs**. F**), and the signal was abolished with primary antibody preincubation with blocking peptide (**N** vs**. J**). Data in **A** and **C** are presented as mean ± SD and analyzed with an unpaired Mann-Whitney test; *n* = 10 water restricted, *n* = 10 water loaded. *P = 0.041, ***P = 0.0005. Original magnification, ×600.

To test whether expression of vasopressin mRNA in the kidney is regulated in vivo, we water-restricted and water-loaded WT mice for 24 hours. We then assessed whole-kidney *Avp* mRNA by qPCR and droplet digital PCR (ddPCR) and protein for preprovasopressin expression and confirmed localization via immunofluorescence. Both vasopressin mRNA and protein were higher in water-restricted versus water-loaded mice (Figure 9, A–C, and Supplemental Figure 4). Consistent with our in vitro data, preprovasopressin protein was found in collecting ducts (Figure 9, D–K). Moreover, collecting duct preprovasopressin signal was higher in water-restricted versus water-loaded mice (Figure 9, F and G vs. J and K; negative control, Figure 9, L–O).

### Vasopressin protein is made by human kidneys.

**Figure 10:**
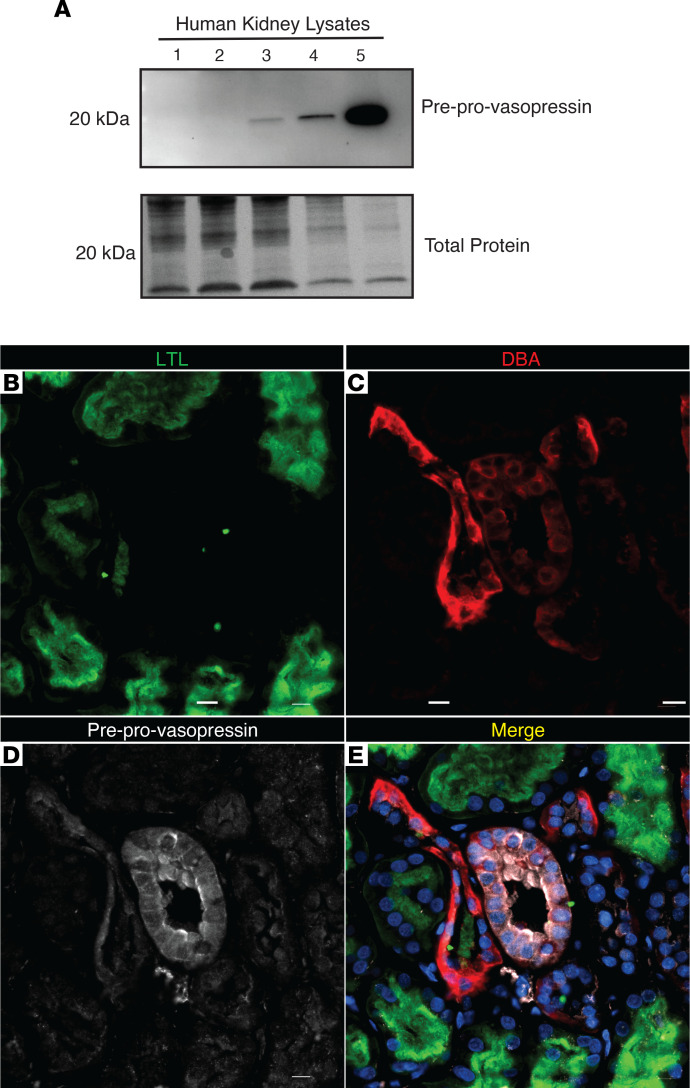
Human kidneys make vasopressin. (**A**) Preprovasopressin was found in healthy human kidney lysates. (**B–E**) Preprovasopressin (white) was found in the collecting ducts (DBA, red) but not in the proximal tubules (LTL, green). Scale bar: 10 μM.

**Figure 11:**
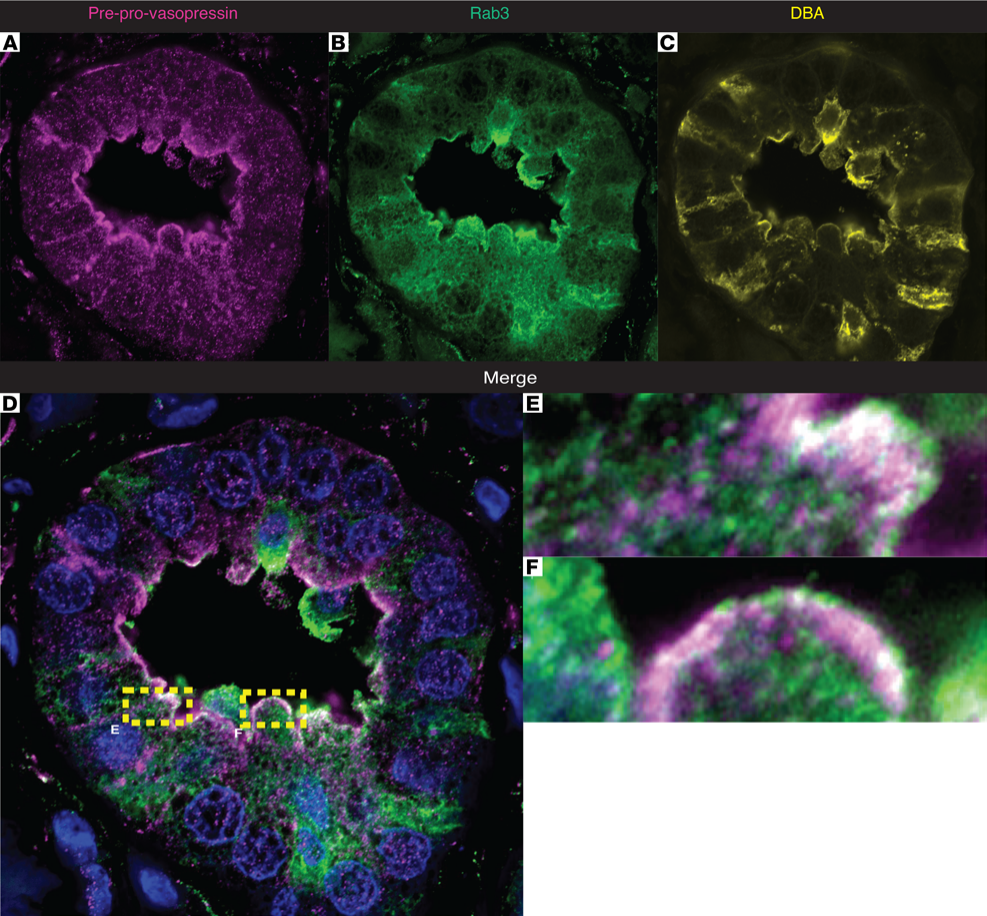
Preprovasopressin colocalizes with Rab3 in human collecting ducts. Superresolution confocal imaging shows that preprovasopressin (pink) colocalizes with Rab3 (green) in DBA-stained (yellow) human collecting ducts. Colocalization is shown in white. Boxed regions in **D** are shown at higher magnification in **E** and **F**. The field in **A–D** is 74 μM × 74 μM and that in **E** and **F** is 8 μM × 4 μM.

Whole-kidney mRNA data (Supplemental Figure 1A) suggested vasopressin expression in human kidneys. Therefore, we obtained samples of frozen and FFPE human kidney tissue to test for vasopressin protein expression. We found that human kidneys could express vasopressin mRNA (Supplemental Figure 4B), which colocalized with *AQP2* mRNA (Supplemental Figure 5) and preprovasopressin protein (Figure 10A). Moreover, preprovasopressin was found in collecting ducts (Figure 10, B–E), and it colocalized with Rab3 in the apical surface of the collecting duct (Figure 11). Together, these data indicate that vasopressin is found in human collecting duct cells in vivo.

## Discussion

Vasopressin is thought to be made primarily in the brain, and that the brain is the sole source of the vasopressin which stimulates vasopressin V2Rs in the kidney. In the present study, we challenge the current dogma. We developed an antibody that recognizes the preprovasopressin peptide to study local production of vasopressin in the kidney. We used this antibody to confirm that preprovasopressin was made in mouse collecting ducts cells in vitro and in vivo. We then showed that kidney-derived preprovasopressin production was responsive to water restriction, which increased mRNA and protein expression in mice. Finally, we also determined that vasopressin mRNA and preprovasopressin protein were found in healthy human kidney collecting ducts. Thus, we provide what we believe to be novel evidence of physiologic, biologically active extrahypothalamic production and regulation of kidney-derived vasopressin from kidney epithelial cells.

Given the prior reports of vasopressin production outside of the brain (21–29), and the publicly available single-cell RNA-Seq data (GSE76632, Supplemental Figure 2; GSE66494, Supplemental Figure 6 and Supplemental Tables 1 and 2), we confirmed vasopressin mRNA in mouse and human kidneys (Figures 1 and 8 and Supplemental Figures 1, 2, and 5) in collecting duct cells. Our reporter mouse further confirmed the expression of *Avp* mRNA in vivo in collecting duct epithelial cells. Although the presence of mGFP could mean *Avp* gene expression occurred in a progenitor cell, our data showing changes in *Avp* mRNA abundance in vivo suggest that there is ongoing *Avp* gene expression in kidney collecting duct cells.

In the hypothalamus, *Avp* can be stimulated by NaCl (40). We found that, in collecting duct cells in vitro, *Avp* was expressed at baseline in vitro and NaCl increased *Avp* mRNA (Figure 2). Interestingly, NaCl is known to increase *Aqp2* independent of exogenous vasopressin, but the contribution of endogenous vasopressin had never been studied (5, 34). We found that V2r was activated in vitro by endogenous vasopressin in response to hypertonic stress. Moreover, the addition of a V2r antagonist prevented hypertonicity-induced increase in *Aqp2* expression in a dose-dependent manner (Figure 2E). Thus, these data suggest that vasopressin is produced by collecting duct cells in vitro and plays a role regulating *Aqp2* expression.

Vasopressin is difficult to quantify (41, 42). Commercially available methods to measure vasopressin, including ELISAs and antibodies, all detect terminally processed peptides, preventing discrimination between local production and peripheral uptake of hypothalamic vasopressin or related peptides. To circumvent this, we developed an antibody that specifically detects the preprovasopressin peptide, as the epitope spans the cleavage regions (Figure 3A). Our antibody detected a band at the expected weight (~20 kDa) in mouse brain and kidney (Figure 3B and Supplemental Figure 3A); it stained regions of the brain known to produce vasopressin (Supplemental Figure 3C) as well as murine IMCDs (Figure 4). Moreover, after immunoprecipitation with our antibody, separate anti-vasopressin and anti-neurophysin 2 antibodies detected a band at the expected weight for preprovasopressin (Supplemental Figure 3, E–H). With this antibody, we were able to determine that immunoreactive preprovasopressin could be found in both mouse and human whole-kidney lysates (Figure 3, C and D, and Figure 10, A and B), localized to the distal nephron, and could be found in Rab3-positive vesicles in mice and humans, which suggests it can be secreted (Figures 5 and 11). To our knowledge, this is the only available method that can detect the vasopressin prepropeptide. This is critical, as peripheral uptake of circulating vasopressin or copeptin could account for nonspecific staining with other methodologies.

We designed a bioassay to assess if the locally produced preprovasopressin was biologically active (Figure 6A) and found that biologically active vasopressin was present in media from IMCDs (Figures 6 and 7). Our bioassay relied on V2r activation by a ligand found in IMCD supernatant to assess the biologic activity of vasopressin. In our model, it possible that luciferase and/or V2R were activated independent of vasopressin. However, the decrease in luciferase signal (Figure 6B, lanes 5 and 6) and AQP2 phosphorylation (Figure 7, M and N) seen with the addition of OPC-31260 suggests that there is a V2R-specific ligand in IMCD medium. Our data do not preclude the possibility that local vasopressin could be signaling through other pathways, including intracellular binding to the V2R (43). Although cancer cell lines have been reported to make vasopressin (44), and there are reports of vasopressin mRNA expression in tissues outside of the hypothalamus (21–29), the production of biologically active vasopressin by nonmalignant cells has not been reported.

Hypertonicity is a known regulator of hypothalamic vasopressin production. We found that both *Avp* mRNA (Figure 9A and Supplemental Figure 4) and preprovasopressin protein (Figure 9, B and C) were higher in kidneys from water-restricted animals compared with those in water-loaded animals. These in vivo observations corroborated our in vitro data and suggest that local vasopressin could be playing a role in urine concentration and dilution in vivo. From these results we can conclude that kidney-derived vasopressin is produced by collecting duct cells in vivo and production is regulated by hypertonic stress.

The physiologic pathways through which hypothalamic vasopressin is involved in regulation of water and blood pressure homeostasis have been known for over 50 years (1). However, there is a large body of literature on the nonwater, non–blood pressure effects of vasopressin, for which physiologic feedback loops have not been clearly defined (7–20). Nonosmotic vasopressin production has been ascribed to hypothalamic stimulation via other mechanisms, e.g., nausea and pain (6, 45), but to our knowledge there is no prior reported data on vasopressin production outside of the brain under physiologic conditions. Interestingly, the vasopressin peptide evolved prior to the development of the vertebrate neurohypophyseal system (46). This finding could explain why cells outside of the neurohypophyseal system express vasopressin. As such, nonmalignancy-associated extrahypothalamic production of vasopressin has also been reported previously in the heart, ovaries, testis, and adrenal glands (21–29). However, these studies did not establish whether this extrahypothalamic vasopressin was biologically active.

Our study extends the current model in which vasopressin is solely produced in the brain under physiologic conditions. Many questions remain regarding the in vivo relevance of our observations. Given that kidney-derived vasopressin is stimulated by hypertonicity and patients with diabetes insipidus have low medullary tonicity, we would expect patients with central or nephrogenic diabetes insipidus to have low-to-absent levels of kidney-derived vasopressin. This might explain why kidney-derived vasopressin is unable to overcome the concentrating defect in patients with nephrogenic diabetes insipidus. Interestingly, V2R stimulation has differing effects depending on whether vasopressin is apical or basolateral, and reports of intracellular activation of the V2R open the door to the possibility that local vasopressin production may regulate V2R from within the cell (43, 47). Moreover, there are data that suggest that kidney-derived vasopressin mRNA is upregulated in humans with CKD (refs. 31, 48; Supplemental Figure 6A) and in mice after kidney injury (ref. 49; Supplemental Figure 6B), implying that the local kidney-vasopressin system could be involved in pathways beyond water homeostasis.

In conclusion, our data show that there is regulated expression of the vasopressin gene in the kidney in both mice and humans and that kidney epithelial cells make biologically active vasopressin in response to NaCl-mediated hypertonicity. It is well known that vasopressin contributes to progression of nondiabetic, diabetic, and polycystic kidney disease (18, 50–52). Our identification of a local vasopressin system in the kidney could provide insight as to how the vasopressin system contributes to kidney function in health and disease.

## Methods

### In silico database analysis

To analyze expression of the AVP gene in a systematic manner, we used Nephroseq v5.0, an online platform hosted by the University of Michigan that allows integrative data mining of publicly available, peer-reviewed genotype/phenotype data (31). We then used the gene name “AVP” and set the following thresholds: *P* = 0.05, *r* = 0.5, and fold change = 1.5. Complete results of the analysis are reported in Supplemental Tables 1 and 2. Publicly available single-cell RNA-Seq data from GSE76632 and GSE66494 were analyzed as normalized expression of reported values in Geo2r (Supplemental Figures 2 and 6).

### Cells

WT C57BL/6J IMCDs were isolated and transfected with SV40 large T cell antigen as described previously (53). Cells were cultured high-glucose and pyruvate-containing DMEM supplemented with 10% FBS and 100 IU/mL penicillin plus 100 (μg/mL) streptomycin and grown to confluency on permeable supports. Cells from passages 2–7 were plated at a density of 5 × 10^5^ cells per well (12-well dish), and sera were withdrawn 24 hours prior to experiments. We confirmed that our IMCDs have a gene expression profile similar to that of native IMCDs, including *Aqp2*, *Aqp3*, *Aqp4*, *Slc14a2*, and *Avpr2* (Supplemental Figure 2A).

#### Hypertonic stimulation.

Confluent IMCDs were serum starved for 24 hours, after which new FBS-free DMEM was supplemented with 100 mmol NaCl in the presence or absence of decreasing doses of V2r inhibitor, OPC-31260. Cells were kept in the NaCl with or without OPC-supplemented medium for 24 hours, after which cells were harvested in Trizol (Invitrogen) for RNA extraction or RIPA (Thermo Fisher Scientific) for total protein extraction. To compare hypertonic stimuli, FBS-free DMEM supplemented with 100 mmol NaCl, 200 mmol glucose, or 200 mmol mannitol was added to serum-starved confluent IMCDs for 12 or 24 hours. Cells were then harvested in Trizol (Invitrogen) for RNA extraction.

#### Conditioned medium.

Confluent IMCDs were serum starved for 24 hours, after which the control group was treated with FBS-free DMEM, and the NaCl group was treated with FBS-free DMEM supplemented with 100 mmol NaCl. After 24 hours the medium was collected from both NaCl-treated and control groups, and the NaCl-conditioned medium was diluted to achieve a calculated osmolality similar to that of the control medium (~320 mOsm) (Figure 6A). The control and NaCl-conditioned media were then added to confluent serum-starved HEK-hV2R-CRE-Luc cells or IMCDs or (see below) for 3 hours. The 3-hour time point was chosen because prior work by Hasler et al. showed that 3 hours of hypertonic stimulation decreased AQP2 expression (34). IMCDs were then harvested in RIPA buffer (Thermo Fisher Scientific) or fixed on a slide with 4% PFA for immunolabeling, and HEK-hV2R-CRE-Luc cells underwent the luciferase reporter assay as described below.

#### V2R luciferase reporter cell assay.

*piggyBac* transposon vectors (54) were designed and ordered from Vectorbuilder to express the hV2R along with puromycin resistance (PB-Puro-CMV-hAVPR2) or cAMP response element driving luciferase (pPB-CREminiP-Luc) in separate vectors. HEK293 cells were transfected in 6-well plates at 60% confluence using Lipofectamine LTX (Invitrogen) with 1 μg of each transposon vector, along with 0.5 μg pCMV-m7PB hyperactive transposase (55), according to the manufacturer’s instructions. One day after transfection, cells were split to 100 mm dishes and selected with 3 μg/ml puromycin for 2 weeks. For luciferase assays, stably transfected cells (HEK-hV2R-CRE-Luc) were treated with different reagents as indicated for 3–18 hours in 24-well plates. Two μl of 30 mg/ml XenoLight D-Luciferin (PerkinElmer) was added to wells after treatment and incubated for 5 minutes. Luciferase expression was quantitated by capturing photons/second using a PerkinElmer IVIS imaging system.

#### HEK293 transient transfection.

HEK293 cells were cultured in 6-well plates to 60% confluence and transfected using Lipofectamine LTX (Invitrogen) using 2.5 μg pCMV6-XL5 or pCMV6-XL5-*AVP* (Origene) following the manufacturer’s instructions. Cells were collected in RIPA buffer (Thermo Fisher Scientific) 24 hours after transfection.

#### siRNA transfection.

IMCDs were plated at a density 7.5 × 10^5^ cells per well. Upon reaching 30% confluency, they were transfected with Silencer Select (Thermo Fisher Scientific) *Avp* siRNA (AssayID s232140) or ctrl siRNA (AssayID 4390843) according to the manufacturer’s instructions. Cells were lysed in RIPA buffer (Thermo Fisher Scientific) upon reaching 90% confluency 24–36 hours later, and the amount of preprovasopressin protein was evaluated with immunoblotting (see below).

### Mice

#### Avp^tm1.1(cre)Hze^; Gt(ROSA)26Sor^tm4(ACTB-tdTomato,-EGFP)Luo^.

Heterozygote B6.Cg-*Avp^tm1.1(cre)Hze^*/J (AVP-IRES2-Cre-D) mice (Jax, 023530), in which Cre-recombinase is linked to arginine vasopressin gene expression (56), were crossed with homozygous B6.129(Cg)-*Gt(ROSA)26Sor^tm4(ACTB-tdTomato,-EGFP)Luo^*/J (ROSA^mT/mG^) mice (Jax, 007676), and a 2-color fluorescent Cre-reporter allele that expresses membrane GFP in Cre-expressing cells (57) to generate the AVP-IRES2-Cre-D;ROSA^mT/mG^ reporter mice. Age-matched, 8-week-old, AVP-IRES2-Cre–positive and –negative ROSAmT/mG-positive mice were used. Genotype was confirmed before and after euthanasia with Transnetyx automated genotyping. Following euthanasia, kidneys were removed and incubated at room temperature overnight in 3.7% formaldehyde, 10 mM sodium m-periodate, 40 mM phosphate buffer, and 1% acetic acid. The fixed kidneys were dehydrated through a graded series of ethanol, embedded in paraffin, sectioned (5 μm), and mounted on glass slides. Slides were then probed with primary antibodies against GFP (Aves Labs, GFP-1020) and goat anti-chicken Alexa Fluor 488 secondary (Thermo Fisher Scientific, A32931) and costained with a mouse anti–AQP2-AF647–conjugated antibody (Santa Cruz, sc-515770) to identify collecting duct cells (Supplemental Figure 1).

### Water loading and restriction

Age-matched male WT C57BL/6J mice (8–12 weeks old) were used for the experiments.

#### Water restriction.

Twenty-four hours prior to euthanasia, water was removed from cages of mice in the water-restricted group.

#### Water loading.

Twenty-four hours prior to euthanasia, food in the water-loaded group was changed to a gelled diet (58). Briefly, 65 g crushed 4.5% fat mouse chow (LabDiets, 5L0D) was mixed with 7 g gelatin and dissolved in 120 mL water. Gel was solidified in plastic cups and then served as the sole source of food for 24 hours, 9:00 AM to 9:00 AM. Ad libitum access to water was maintained throughout.

### Reverse transcription and real time qPCR

RNA from cells and kidneys was isolated with Trizol reagent (Invitrogen) following the manufacturer’s instructions. cDNA was synthesized from equal amounts of total RNA from each sample using the SuperScript IV First-strand Synthesis System kit (Invitrogen).

#### Reverse transcription.

PCR was performed using Q5 High-Fidelity DNA Polymerase (New England BioLabs): forward primer, ATGCTCGCCAGGATGCTCAACACTACG, and reverse primer, TCAGTAGACCCGGGGCTTGGCAGAATCCACGGACTC.

#### Quantitative RT-qPCR.

Quantitative RT-qPCR was carried out using TaqMan real-time PCR (Applied Biosystems, 7900HT). All gene probes and master mix were purchased from Applied Biosystems. The probes used in the experiments were as follows: mouse *Avp* (Mm00437761_g1) (Figure 2) and (Mm01271704_m1) (Supplemental Figure 2D) as well as mouse *Aqp2* (Mm00437575_m1), mouse *Aqp3* (Mm01208559_m1), mouse *Aqp4* (Mm00802131_m1), mouse *Akr1b3* (Mm01135578_g1), mouse *Slc14a2* (Mm01261839_m1), mouse *Avpr2* (Mm01193534_g1), and mouse RPS18 (Mm02601777).

### ddPCR

All ddPCR experiments were performed using a QX200 AutoDG Droplet Digital PCR System (Bio-Rad) with assistance from the Vanderbilt University Medical Center Immunogenomics, Microbial Genetics, and Single Cell Technologies Core. For all experiments, plates of droplets of PCR mixture were automatically generated with an AutoDG (Bio-Rad), TaqMan probes (Thermo Fisher) were added, and the plates were heat sealed and amplified with a C1000 Touch Thermal Cycler (Bio-Rad). Droplets were then read with a Droplet Reader (Bio-Rad). DNA concentrations were determined only from wells with more than 12,000 droplets using QuantaSoft v1.7.4.0917 (Bio-Rad) after manually setting the positive droplet threshold above the signal from no-template controls in the same plate. Counts are reported as copies per 20 μL normalized to Tert gene expression. The following probes were used: mouse *Avp* (Mm00437761_g1), human *AVP* (Hs00356994_g1), and TaqMan Copy Number Reference Assay (mouse and human, Tert 4458368 and 4403316).

### RNA in situ hybridization

Experiments were performed with RNAScope reagents (ACD, Biotechne) and according to the manufacturer’s instructions. Briefly, FFPE kidney sections (mouse and human) were stained using the RNAScope Multiplex Fluorescent Reagent v2 kit RED (Advanced Cell Diagnostics Inc.). Probes against mouse vasopressin (Mm-*Avp*; 472261) mouse aquaporin 2 (Mm-*Aqp2*; 452411), human vasopressin (Hs-*AVP*; 401361), and human aquaporin 2 (Hs-*AQP2*; 434861) were used, and the 3-Plex negative control probe provided by the manufacturer was used as a negative control.

### Antibody development

Antibody was developed with assistance from Phosphosolutions Inc. Briefly, rabbits were immunized with a synthetic peptide that corresponds to amino acid residues that span the cleavage sites between vasopressin and neurophysin 2, which were conjugated to keyhole limpet hemocyanin. Antibodies were then purified with affinity purification.

### Immunoblotting

Protein was extracted from cells and whole kidneys using RIPA buffer (Thermo Fisher Scientific) with protease and phosphatase inhibitors (Roche), total protein was then quantified with BCA Assay (Pierce), and equal amounts of protein were loaded in MiniProteanTGX polyacrylamide precast gels (Bio-Rad) and transferred to nitrocellulose using Transblot Turbo (Bio-Rad). Nitrocellulose membranes were stained for total protein using Ponceau-S (Thermo Fisher Scientific) and blocked with 5% nonfat dry milk for 1 hour at room temperature. Primary antibodies used were as follows: anti-preprovasopressin (PhosphoSolutions), 1:1,000 overnight; anti-aquaporin E2 (Santa Cruz, sc-515770), 1:100 overnight; and anti-aquaporin phosphor serine 269 (59) (Phosphosolutions, p112-269), 1:1,000 overnight. Secondary antibodies were HRP-coupled anti-mouse (AB_10015289) and anti-rabbit (AB_2337938) from Jackson ImmunoResearch. Band density quantification was performed with ImageJ (NIH).

### Immunoprecipitation

Magnetic protein G beads (Millipore-Sigma) were used per the manufacturer’s instructions. Briefly, protein G beads were coupled to the preprovasopressin antibody or normal rabbit serum (Millipore-Sigma) at a 1:50 ratio for 10 minutes at room temperature, after which 1 mg of IMCD whole-cell lysate was added to the antibody-coupled beads and incubated for 1 hour at room temperature. Proteins were then eluted, mixed with 2× Laemmli buffer (Bio-Rad), run on polyacrylamide gels, transferred to nitrocellulose membranes, and incubated with anti-preprovasopressin (PhosphoSolutions, 1:1,000) for 1 hour at room temperature, anti-vasopressin (Millipore-Sigma, AB 1565; 1:1,000) (60, 61) for 1 hour at room at temperature, or anti-neurophysin 2 (Millipore-Sigma, MABN856) (62) followed by HRP-coupled anti-rabbit (AB_2337938) or anti-mouse (AB_2313585) from Jackson ImmunoResearch 1:10,000 for 1 hour at room temperature.

### Immunofluorescence

#### Cells.

IMCDs were plated on Corning 6-well 0.4 μM pore Transwell inserts or Nunc-Lab Tech II Chamber Slides (Thermo Fisher Scientific) and grown to confluency. Cells were then stimulated with isotonic FBS-free control or isotonic FBS-free NaCl-conditioned medium per above. After 3 hours of stimulation, cells were washed in ice-cold PBS, fixed with 4% PFA for 30 minutes at room temperature, blocked with 5% BSA in PBS 0.2% Tween for 1 hour at room temperature, and probed with anti-aquaporin E2 FITC conjugated (Santa Cruz, sc-515770; 1:100) overnight, anti-preprovasopressin (PhosphoSolutions, 1:1,000) for 1 hour at room temperature, anti-Rab3a (Thermo Fisher Scientific, MA5-27162; 1:100) for 1 hour at room temperature, and ActinRed555 (Life Technologies) per the manufacturer’s protocol. Cells were imaged on a Nikon TiE fully motorized inverted fluorescent microscope or a Zeiss LSM 980 Confocal AiryScan 2.

#### Kidneys.

Following euthanasia, kidneys were removed and incubated at room temperature overnight in 3.7% formaldehyde, 10 mM sodium m-periodate, 40 mM phosphate buffer, and 1% acetic acid. The fixed kidney was dehydrated through a graded series of ethanol, embedded in paraffin, sectioned (5 μm), and mounted on glass slides. Immunostaining was carried out as described previously (63). Primary antibodies used are as follows: anti-preprovasopressin (PhosphoSolutions, 1:1,000) for 1 hour at room temperature, anti-aquaporin E2 FITC conjugated (Santa Cruz, sc-515770; 1:100) overnight, anti-Rab3a (Thermo Fisher Scientific, MA5-27162; 1:100) for 1 hour at room temperature, or Dolichos Biflorus Agglutinin (DBA), Rhodamine labeled (Vector) and Lotus Tetragonolobus Lectin (LTL) — Fluorescein labeled (Vector) or ActinRed 555 ReadyProbes (Thermo Fisher Scientific). Secondary antibodies used are as follows: goat anti-Rabbit AF647 coupled (Invitrogen, A27040) and goat anti-Mouse Alexa Fluor 488 coupled (Invitrogen, A11001). 3D images were assembled using the ImageJ 3D viewer.

### Statistics

All values are shown as mean ± SD. Between-group comparisons were made using 1-way ANOVA with Tukey’s post hoc test or Mann-Whitney test as indicated in figure legends. *P* < 0.05 was used as the significance threshold. Analysis was performed using Prism 9 software (GraphPad).

### Study approval

All human tissue samples were obtained in accordance with and following approval by the Vanderbilt University Medical Center IRB. Human tissue samples were obtained as unidentified frozen human tissue, procured through the Collaborative Human Tissue Network, and the FFPE deidentified blocks were procured through the biorepository of the Vanderbilt University Medical Center’s Department of Pathology.

All animal experiments were performed in accordance with the guidelines of and with the approval of the Institutional Animal Care and Use Committee of Vanderbilt University Medical Center.

## Author contributions

JPA, GB, MHW, and RCH conceived the study and designed experiments. JPA, YZ, CM, FB, MJ, RMV, MEK, WL, and JC performed experiments. JPA, AST, FB, JAW, ERG, AXM, RMV, MEK, RZ, EH, MHW, GB, MZ, and RCH analyzed data. JPA, AST, EH, RZ, GB, and RCH wrote and edited the manuscript.

## Supplementary Material

Supplemental data

## Version 1 11/03/2022

In-Press Preview

## Version 2 12/22/2022

Electronic publication
