# Supplementary material for: Kidney collecting duct cells make vasopressin in response to NaCl-induced hypertonicity
Source: JCI Insight. 2022 Dec 22;7(24):e161765. doi: 10.1172/jci.insight.161765 (PMC9869977; doi:10.1172/jci.insight.161765)
Supplement: Supplemental data [file jciinsight-7-161765-s097.pdf]

Supplemental Figure 1

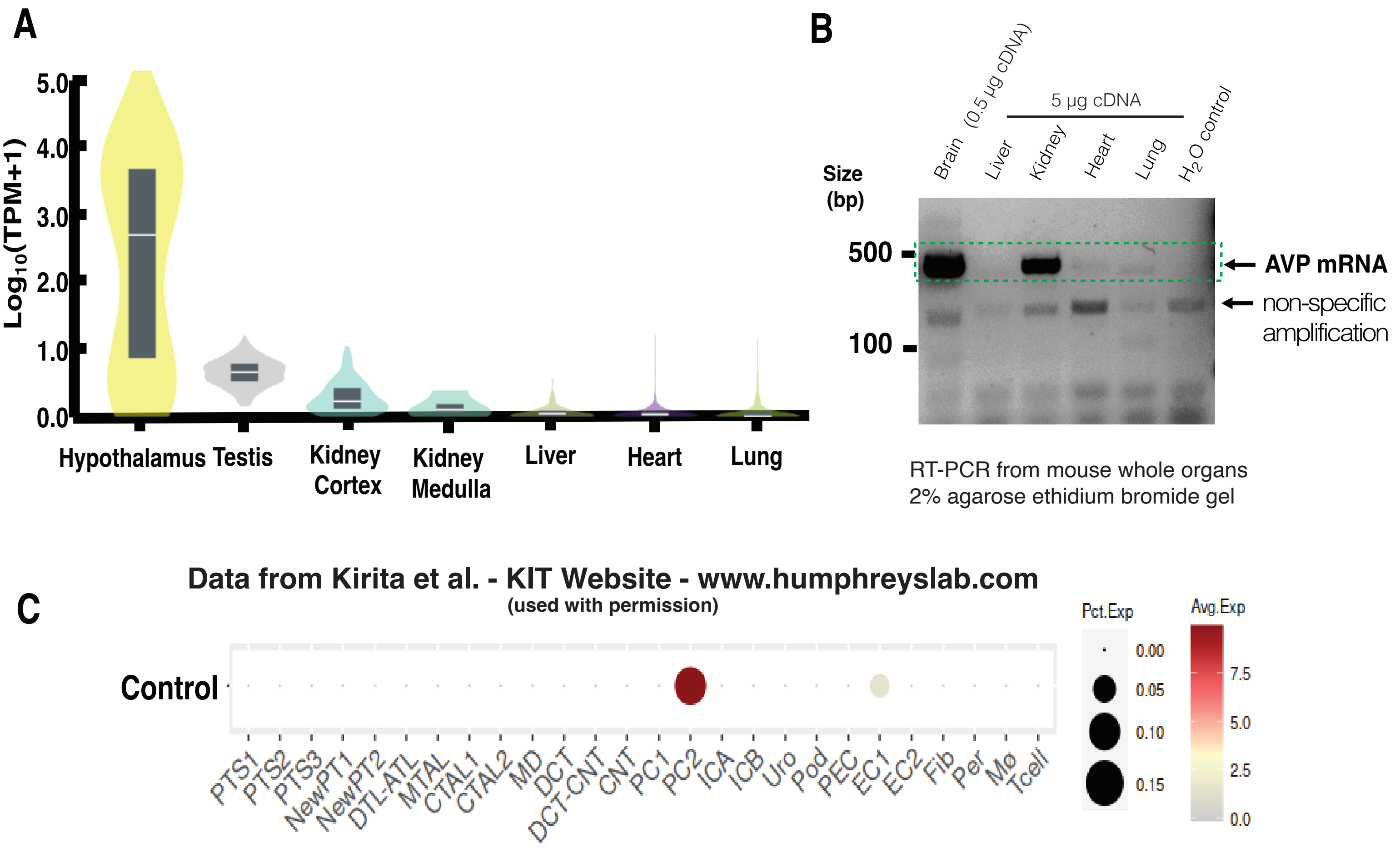

Supplemental Figure 2

Data from Schulze Blasum et al. - GSE76632

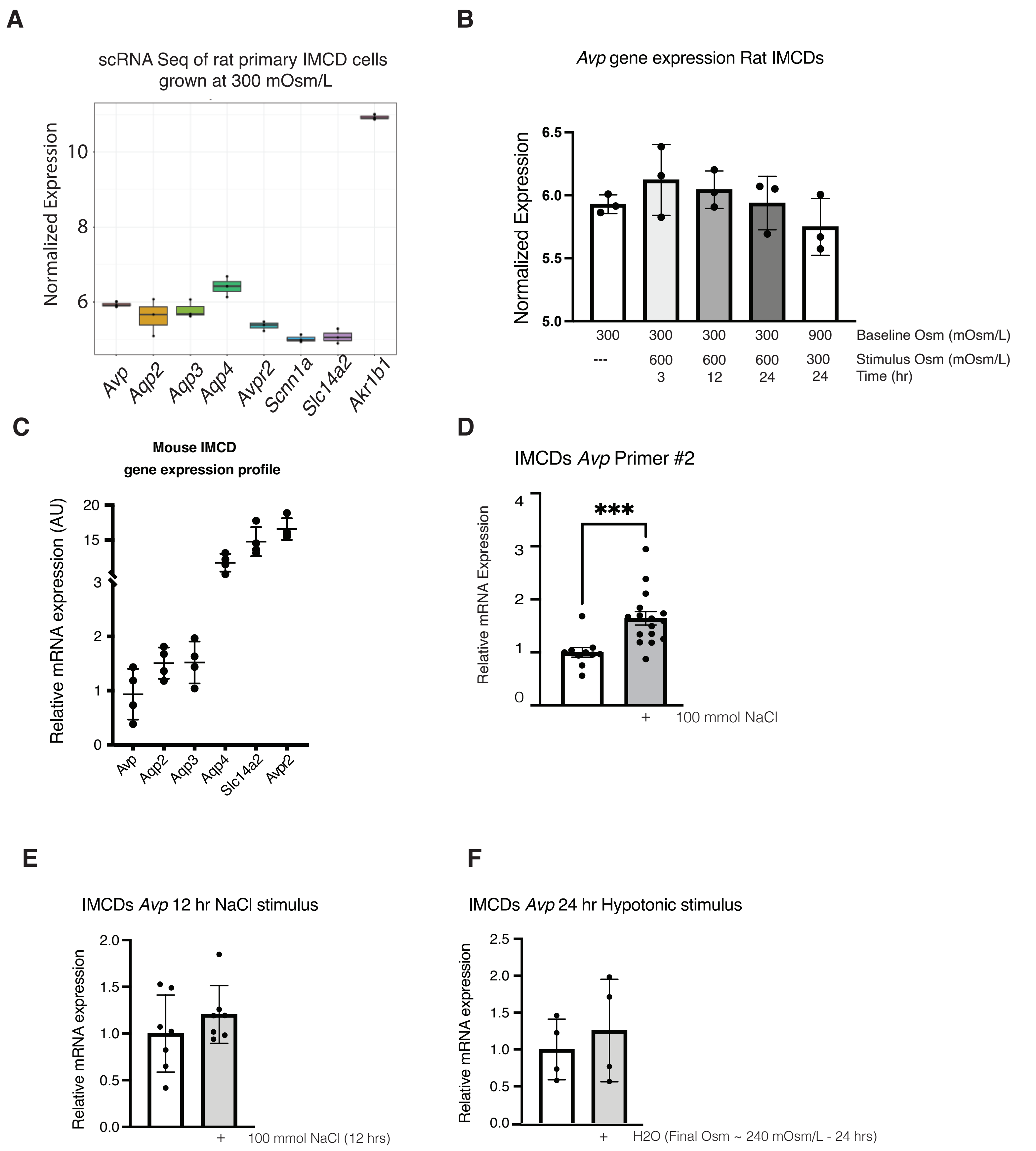

Supplemental Figure 3

Characterization of the pre-pro-vasopressin antibody

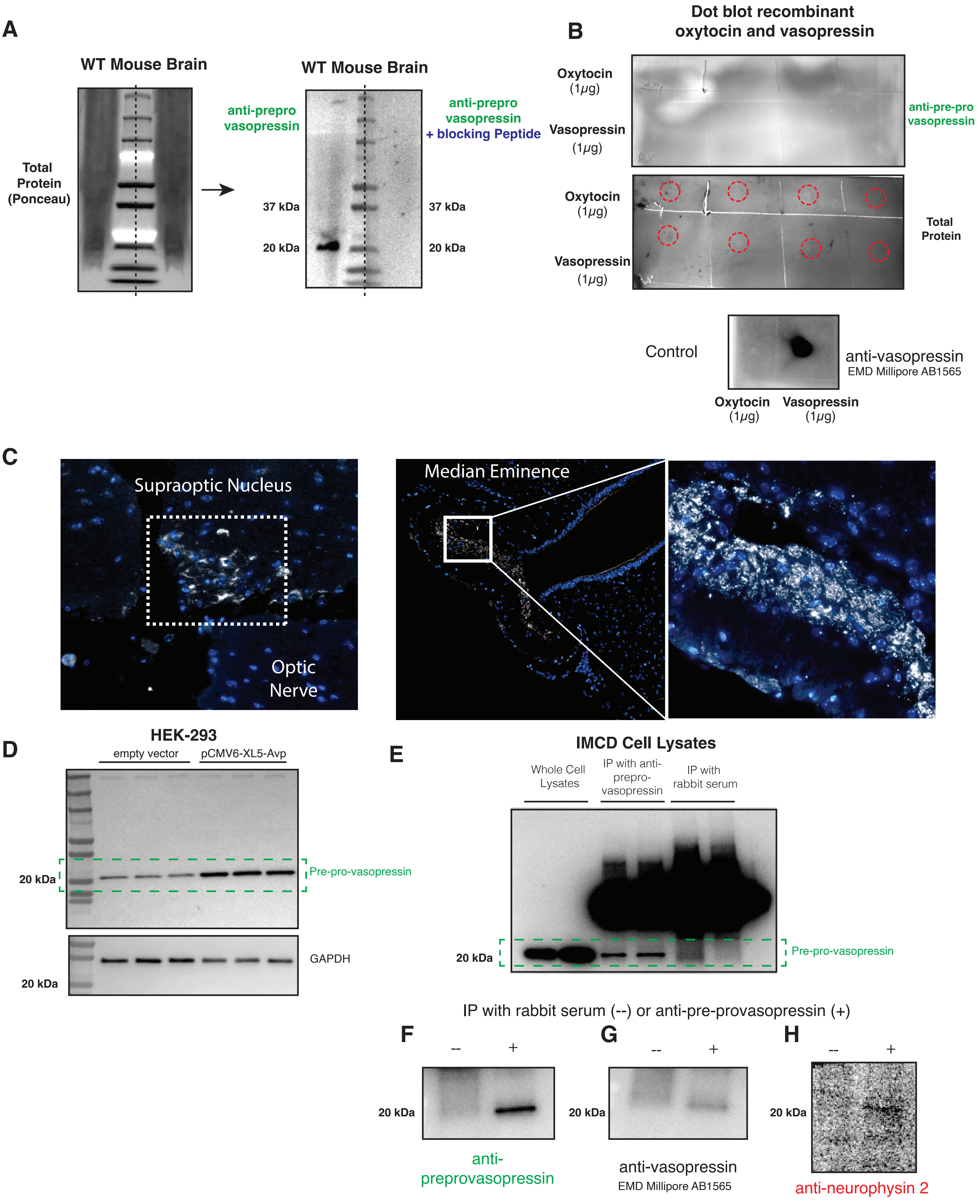

Supplemental Figure 4

A

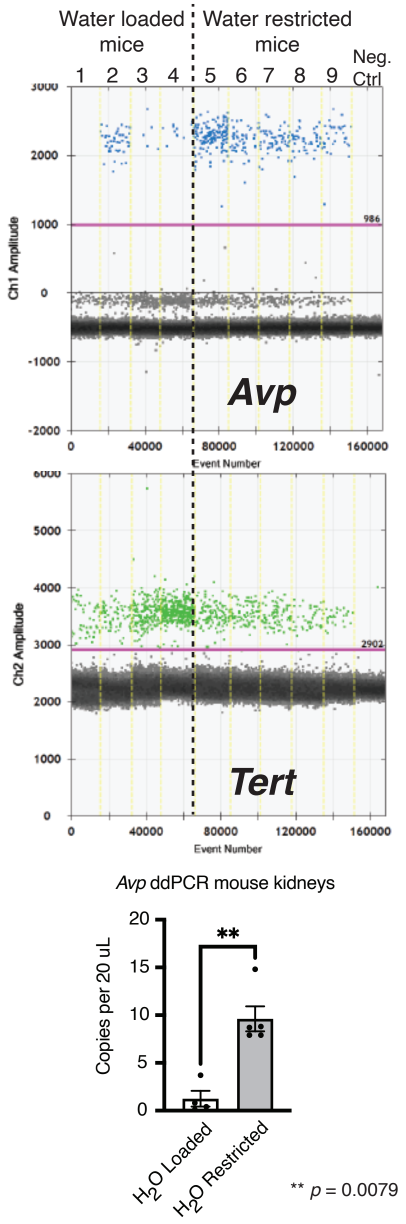

B

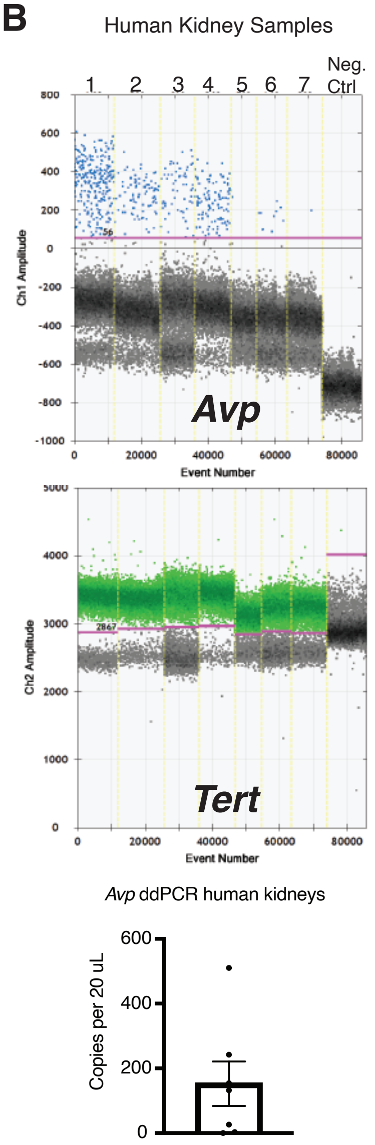

Supplemental Figure 5

***AVP* mRNA co-localizes with *AQP2* mRNA in human collecting ducts**

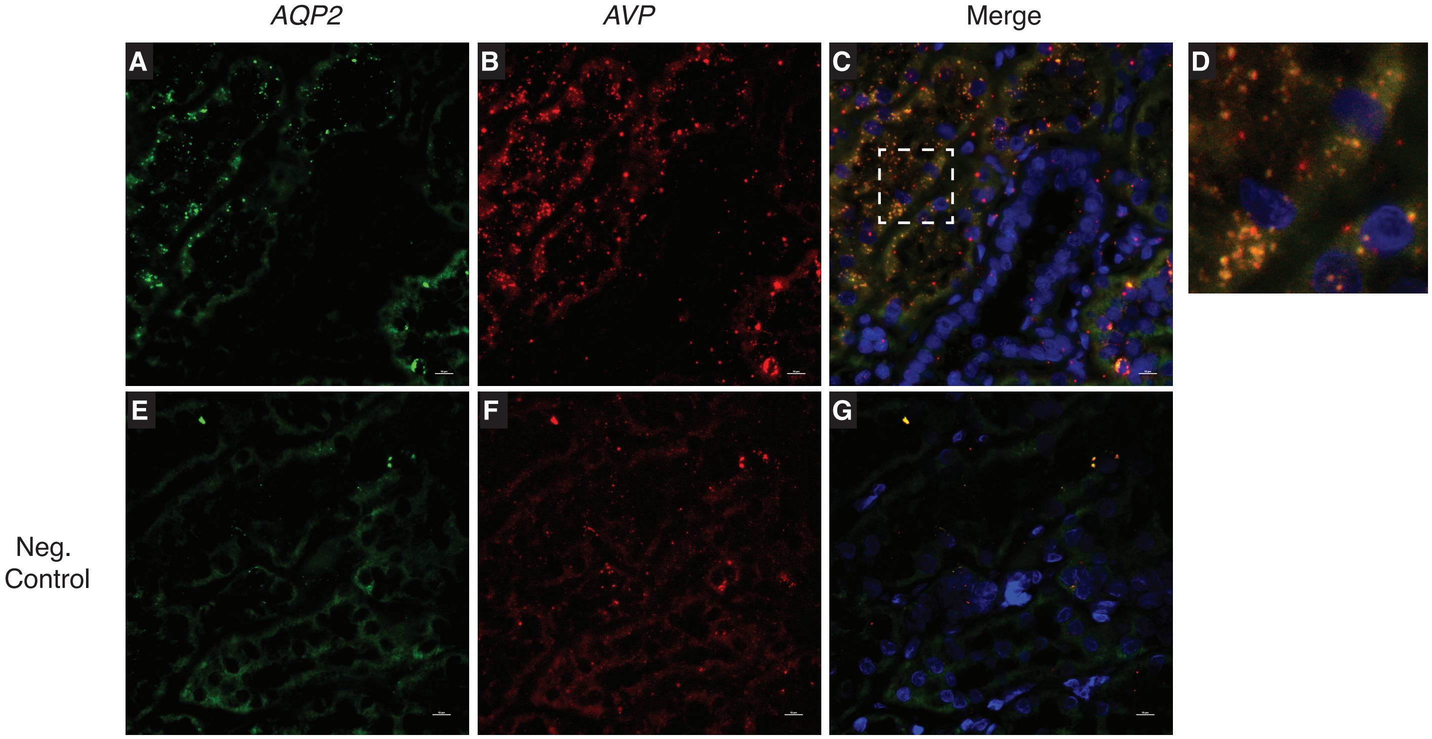

Supplemental Figure 6

A

Human kidney RNAseq
Nakagawa et al. dataset - GSE66494

Kidney vasopressin mRNA

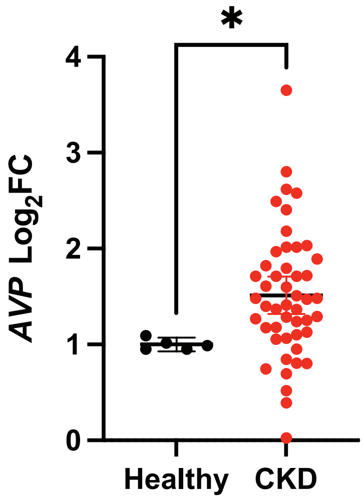

* $p = 0.0240$

B

Data from Kirita et al. - KIT Website - www.humphreyslab.com
(used with permission)

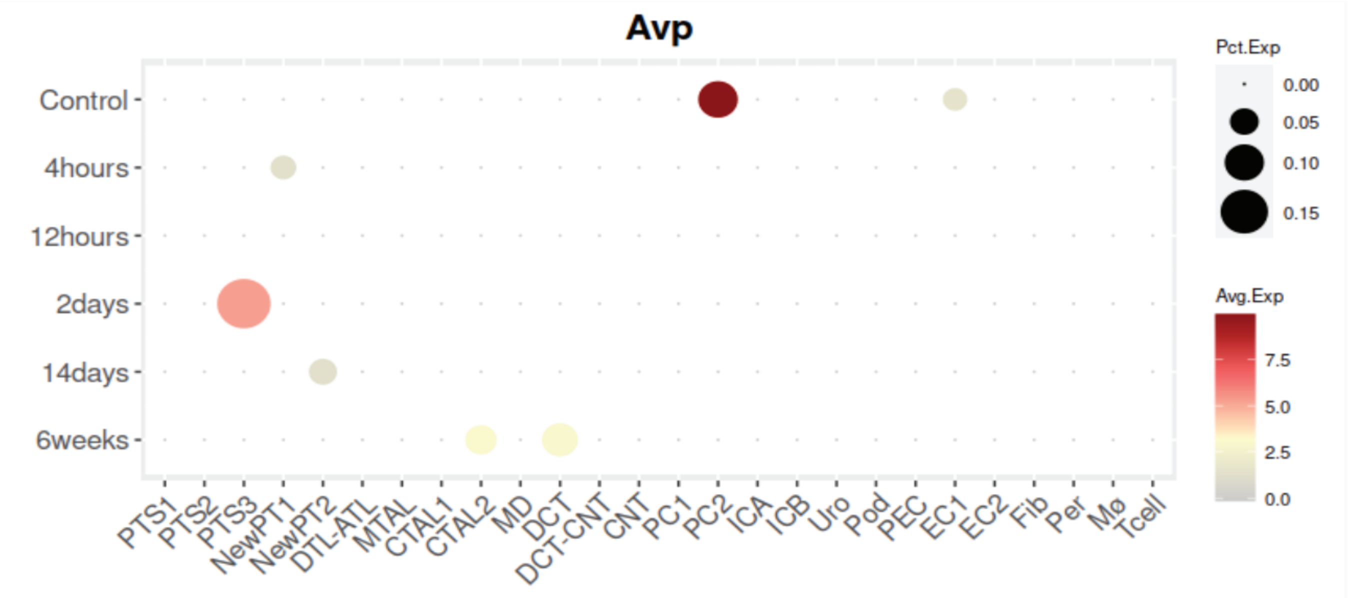

Supplementary Table 1

Summary of Analyses for AVP		
Significance based on thresholds: p Value: 0.050; r Value: 0.5; Fold change: 1.5		
**Analysis Type**	**Significant Analyses**	**Total Unique Analyses**
GFR Analyses	6	43
Serum Creatinine Analyses	3	47
Age Analyses	2	44
Disease vs. Control Analyses	2	41
Proteinuria Analyses	2	46
Transplant Analyses	2	11
Blood Pressure Analyses	1	23
Body Mass Index Analyses	1	25
BUN Analyses	1	5
Fasting Blood Glucose Analyses	1	4
erstitial Fibrosis and Tubular Atrophy Analyses	1	2
ACR Analyses	0	4
APOL1 Risk Status Analyses	0	7
Disease vs. Other Disease(s) Analyses	0	51
Donor Type Analyses	0	2
Hemoglobin Analyses	0	0
Mesangial Hypercellularity Analyses	0	1
Post-Event Treatment Analyses	0	1
Race Ethnicity Analyses	0	30
Schena Grade Analyses	0	1
Segmental Glomerulosclerosis Analyses	0	1
Sex Analyses	0	46
Tissue Type Analyses	0	34
Weight Analyses	0	32
WHO Lupus Nephritis Class Analyses	0	6
Total	22	507

Data obtained from NephroSeq v.5.0 www.nephroseq.org, September 2022, University of Michigan, Ann Arbor, MI

Supplementary Table 2

Analyses for AVP
Significance based on thresholds: p Value: 0.050; r Value: 0.5; Fold change: 1.5
Applied filters: no filters applied

Data Type	Dataset	Analysis	Analysis Synopsis	Analysis Type	p-Value	Fold Change	r Value	Reporter
mRNA	Nakagawa CKD Kidney	Chronic Kidney Disease vs. Normal Kidney (Discovery Set)	Disease vs. Control Analysis	over expression	4.60E-06	1.807		A_23_P109133
mRNA	Flechner Transplant Blood	Renal Dysfunction vs. No Rejection (Cadaveric Donors)	Transplant Analysis	under expression	1.30E-04	-1.576		34020_at
mRNA	Woroniecka Diabetes TubInt	(All Measured Samples)	GFR (MDRD) Analysis	correlation	0.003		0.598	207848_at
mRNA	Hodgin FSGS Glom	(Focal Segmental Glomerulosclerosis Samples)	Interstitial Fibrosis and Tubular Atrophy Analysis	correlation	0.003		0.885	g13259532_3p_at
mRNA	Reich IgAN TubInt	(IgA Nephropathy Samples)	Proteinuria Analysis	correlation	0.003		-0.623	207848_at
mRNA	Flechner Transplant Blood	(Cadaveric Donors)	GFR (MDRD) Analysis	correlation	0.004		0.679	34020_at
mRNA	ERCB Nephrotic Syndrome TubInt	(Focal Segmental Glomerulosclerosis Samples)	GFR (MDRD) Analysis	correlation	0.005		0.66	ENSG00000101200
mRNA	Hodgin FSGS Glom	(Focal Segmental Glomerulosclerosis Samples)	Serum Creatinine Analysis	correlation	0.006		0.863	g13259532_3p_at
mRNA	Gunther Transplant Blood	(Acute Rejection Samples)	Age Analysis	correlation	0.007		0.583	207848_at
mRNA	ERCB Lupus TubInt	(Lupus Nephritis Samples)	BUN Analysis	correlation	0.008		-0.886	ENSG00000101200
mRNA	Kurian Transplant Kidney	Laparoscopic Donor Nephrectomy vs. Open Donor Nephrectomy (All Measured Sa	Transplant Analysis	over expression	0.008	2.803		207848_at
mRNA	Flechner Transplant Blood	(Cadaveric Donors)	Serum Creatinine Analysis	correlation	0.01		-0.621	34020_at
mRNA	Cox IgAN Blood	(IgA Nephropathy Samples)	GFR (CG) Analysis	correlation	0.017		0.801	ILMN_1811443
mRNA	Sampson Nephrotic Syndrome TubInt	(Minimal Change Disease Samples)	Proteinuria Analysis	correlation	0.026		-0.665	551
mRNA	Cox IgAN Blood 2	IgA Nephropathy vs. Healthy Living Donor	Disease vs. Control Analysis	over expression	0.037	1.739		207848_at
mRNA	Ju CKD Glom	(Minimal Change Disease Samples)	Serum Creatinine Analysis	correlation	0.037		-0.605	551
mRNA	ERCB Lupus TubInt	(Lupus Nephritis Samples)	GFR (CG) Analysis	correlation	0.038		0.782	ENSG00000101200
mRNA	Flechner Transplant Blood	(Cadaveric Donors)	Age Analysis	correlation	0.042		0.513	34020_at
mRNA	Berthier Lupus TubInt	(Healthy Living Donors)	Blood Pressure Analysis	correlation	0.046		-0.997	207848_at
mRNA	Hodgin Diabetes Mouse Glom	(Diabetic Nephropathy Mouse Model eNOS-deficient C57BLKS db/db)	Fasting Blood Glucose Analysis	correlation	0.046		0.762	11998
mRNA	ERCB Nephrotic Syndrome TubInt	(Focal Segmental Glomerulosclerosis Samples)	GFR (CKD-EPI) Analysis	correlation	0.046		0.641	ENSG00000101200
mRNA	Ju CKD Glom	(IgA Nephropathy Samples)	Body Mass Index Analysis	correlation	0.049		0.516	551

Data obtained from NephroSeq v.5.0 www.nephroseq.org, September 2022, University of Michigan, Ann Arbor, MI
